# Exopolysaccharide is required for motility, stress tolerance, and plant colonization by the endophytic bacterium *Paraburkholderia phytofirmans* PsJN

**DOI:** 10.3389/fmicb.2023.1218653

**Published:** 2023-08-21

**Authors:** Benzhong Fu, Qing Yan

**Affiliations:** Plant Sciences and Plant Pathology Department, Montana State University, Bozeman, MT, United States

**Keywords:** plant endophytic bacteria, plant colonization, *Camelina sativa*, *Pisum sativum*, drought stress

## Abstract

*Paraburkholderia phytofirmans* PsJN is an endophytic bacterium and has been shown to promote the growth and health of many different plants. Exopolysaccharide (EPS) plays important roles in plant-bacteria interaction and tolerance to environmental stresses. However, the function of EPS in PsJN and its interaction with plants remain largely unknown. In this study, a deletion mutation of *bceQ* gene, encoding a putative flippase for the EPS biosynthesis, was introduced in the genome of PsJN. The Δ*bceQ* mutant produced a significantly lower level of EPS than the wild type strain in culture media. Compared to the wild type PsJN, the Δ*bceQ* mutant was more sensitive to desiccation, UV damage, salt (NaCl) and iron (FeCl_3_) stresses, and bacteriophage infection. More importantly, the mutation of *bceQ* decreased the endophytic colonization of PsJN in camelina (*Camelina sativa*) and pea (*Camelina sativa*) under plant drought stress conditions. To the best of our knowledge, this is the first report that EPS production is required for the maximal colonization of an endophytic bacterium in the plant tissues under stress conditions.

## Introduction

Endophytic bacteria colonize the interior sites of plants and promote plant growth and health. Successful plant colonization is necessary for the endophytes to provide benefits (Kandel et al., 2017). Understanding the molecular mechanisms of how endophytic bacteria colonize plants can help us better use the beneficial microorganisms to improve plant yield.

To successfully colonize plant interior tissues, the bacteria need to have an effective epiphytic colonization which includes adhesion and survival on plant surfaces, and an effective endophytic colonization which includes entry through openings and proliferation in the host tissues (Kandel et al., 2017). The surface attachment and epiphytic survival require bacterial flagella, fimbriae, and cell surface polysaccharide (Afzal et al., 2019). Some endophytic bacteria produce cell wall-degrading enzymes including cellulase, xylanases, and pectinase to modify plant cell walls and facilitate bacterial entry (Kandel et al., 2017). Compared to the intensive studies of bacterial adhesion on plant surfaces and entry of plant tissues, relatively limited is known of how the endophytes grow and survive in the plant tissues, especially under stress conditions.

Endophytic bacterium *Paraburkholderia phytofirmans* PsJN (previously called *Pseudomonas* sp. PsJN and *Burkholderia phytofirmans* PsJN) was isolated from onion roots (Frommel et al., 1991) and can colonize different plants including *Arabidopsis thaliana* (Timmermann et al., 2017), potato (Frommel et al., 1991), grapevine (Ait Barka et al., 2006), wheat (Naveed et al., 2014), maize and tomato (Mitter et al., 2013) and quinoa (Yang et al., 2020). It was shown that PsJN promoted plant growth under various abiotic stresses such as drought on wheat (Naveed et al., 2014) and maize (Naveed et al., 2015), salinity on quinoa (Yang et al., 2020), high temperature (32°C) on tomato (Issa et al., 2018), low temperatures (4°C) on grapevine (Ait Barka et al., 2006), and freezing temperatures on *A. thaliana* (Su et al., 2015). Further, PsJN protected plants from biotic stresses caused by pathogenic microorganisms such as the bacterial pathogens *Pseudomonas syringae* on *A. thaliana* (Timmermann et al., 2017) and *Xylella fastidious* on grapevine (Baccari et al., 2019); and fungal pathogens including *Verticillium dahliae* on tomato (Sharma and Nowak, 1998) and *Botrytis cinerea* on grapevine (Miotto-Vilanova et al., 2016). Overall, PsJN can colonize and benefit a wide range of plants and has been established as a model to study the molecular mechanisms of plant-endophytic bacteria interactions (Esmaeel et al., 2018).

Bacterial exopolysaccharides (EPS) are extracellular polymeric substances that play important roles in surface attachment, stress tolerance (Bhagat et al., 2021), and plant-endophytic bacteria interactions. For example, EPS facilitated root attachment and nodule formation on common bean by the nitrogen-fixing bacterium *Paraburkholderia phymatum* (Liu et al., 2020) and was required for tolerance to desiccation and salt stress, and rice colonization by the endophytic bacterium *Gluconacetobacter diazotrophicus* (Meneses et al., 2011; Velázquez-Hernández et al., 2011). Further, the endophytic bacterium *Bacillus safensis* increased its EPS production under heat stress (Mukhtar et al., 2023). Additionally, EPS was found to protect cells of *Bacillus marcorestinctum* from damages caused by ultraviolet radiation (Li et al., 2022).

The genome of PsJN has an EPS biosynthesis gene cluster that contains a total of 19 genes (*bceABCDEFGHIJKNVOPQRST*) that were proposed to make cepacian, the most common EPS produced by species of the *Burkholderia* genus (Weilharter et al., 2011). Cepacian biosynthesis initiates by the formation of nucleotide sugar precursors and is followed by the assembly of oligosaccharide repeat-unit that requires enzymes BceABCGHJKNRT. The assembled oligosaccharide repeat-units are coupled to lipid carriers and translocated, by the putative flippase BceQ, into the periplasmic spaces where the lipid carrier-linked oligosaccharides are polymerized and exported *via* the putative polymerase BceI and the transmembrane proteins BceDEF (Ferreira et al., 2011). Although the cepacian EPS biosynthesis genes were annotated in PsJN, their functions have not been validated. Further, the role of EPS in plant-PsJN interaction remains largely unknown. A recent transcriptomic analysis revealed that expression of the cepacian biosynthesis genes of PsJN was upregulated when the host plant potato was under drought stress (Sheibani-Tezerji et al., 2015). This report is interesting and suggests that PsJN can sense the plant stress signals and adjusts the expression of specific genes, including the EPS biosynthesis genes, to adapt to changes in the plant interior environment caused by plant stress. However, the genetic evidence that EPS plays a role in the endophytic colonization of bacteria in response to plant stress is still missing.

In this study, we aimed to investigate the cepacian EPS biosynthesis genes of PsJN and understand the roles of EPS production in the bacterial traits that influence plant colonization. A PsJN mutant which has a mutation of the EPS biosynthesis gene *bceQ* and lacks the EPS production was generated. The impacts of EPS deficiency on bacterial motility, tolerance to biotic and abiotic stresses, and plant colonization under normal growth and drought stress conditions were characterized.

## Materials and methods

### Plasmids, primers, strains, and culture conditions

The bacterial strains and the plasmid constructs used in this study were listed in Table 1. *P. phytofirmans* PsJN and its derivatives were cultured in KB (King’s B medium), LB (Luria Broth), ½ (half strength) PDA/PDB (Potato Dextrose Agar/Broth, Difco™ USA), NA (Nutrient Agar), R2A or TSA (Tryptone Soya Agar) at 28°C.

**Table 1:** Bacterial strains, plasmids, and primers used in this study.

Strains, plasmids	Characteristics*	Source
*P. phytofirmans*		
LK545	Wild type PsJN, originally isolated from onion roots.	Frommel et al. (1991)
LK583	Spontaneous rifamycin-resistant mutant of the wild type PsJN (LK545), Rif^r^	This study
LK749	Wild type PsJN (LK583) containing the empty vector pME6010	This study
LK754	Δ*bceQ* mutant of PsJN, made by introducing the deletion construct pEX18TC-Δ*bceQ* into wild type PsJN (LK583), Rif^r^	This study
LK750	The Δ*bceQ* mutant (LK754) containing the empty vector pME6010, Rif^r^, Tc^r^	This study
LK751	The Δ*bceQ* mutant (LK754) containing the complementation construct pME6010-*bceQ*full, Rif^r^, Tc^r^	This study
Plasmids		
pEX18Tc	Gene replacement vector with molecular cloning sites from pUC18, *sac*B^+^, Tc^r^	Hoang et al. (1998)
pEX18TC-ΔbceQ	pEX18TC containing a deleted gene *bceQ*. Tc^r^	This study
pME6010	pACYC177-pVS1 shuttle vector. Tc^r^	Heeb et al. (2000)
pME6010-bceQfull	pME6010 containing *bceQ* gene cloned from the wild type PsJN. Tc^r^	This study
Oligo DNA primers	DNA sequences (5′-3′)	
bceQ-up-F	GTAAAACGACGGCCAGTGTTCACGGAACTGCATCGC	This study
bceQ-up-R	GCAGTGAACGCGCAAGATCTACGTTCTTGAGAATGCC	This study
bceQ-down-F	GGCATTCTCAAGAACGTAGATCTTGCGCGTTCACTGC	This study
bceQ-down-R	CAGGAAACAGCTATGACCTTGGTCAGCTTCGACTGC	This study
18Tc-bceQ-F	GCGATGCAGTTCCGTGAACACTGGCCGTCGTTTTAC	This study
18Tc-bceQ-R	GCAGTCGAAGCTGACCAAGGTCATAGCTGTTTCCTG	This study
bceQ-Full-F	TATCCGCTCGAGGAAGCCTTGATGAACGAAGC	This study
bceQ-Full-R	TATCCCAAGCTTCATGCGCTTTTCCTCGGA	This study

*Rif^r^: rifamycin resistance at 100 mg/ml; Tet^r^: tetracycline resistance at 20 mg/ml.

### Construction of PsJN mutant and complementary strain

The in-frame deletion of *bceQ* in the genome of PsJN was performed by following a previous method (Yan et al., 2017). Briefly, a deletion construct pEX18TC-Δ*bce*Q which contains 500 bp of upstream and 506 bp of downstream sequences flanking the *bce*Q gene, was amplified by PCR using primers *bce*Q-up-F and *bce*Q-up-R, *bce*Q-down-F, and *bce*Q-down-R (Table 1), respectively. The deletion construct was transformed into wild-type PsJN and the transformants were cultured on KB plates amended with tetracycline at 20 μg/ml to select colonies that had the deletion construct integrated into the bacterial genome. The transformants were then cultured on KB plates plus 5% sucrose to identify colonies that have the deletion mutation by PCR analysis. Specifically, the wild type PsJN produced a 1,540 bp-DNA fragment and the Δ*bceQ* mutant produced a 163 bp-DNA fragment, respectively (Supplementary Figure S1), using the primers *bceQ*-Full-F and *bceQ*-Full-R (Table 1).

The complementary strain Δ*bceQ*-Comp (LK751) was generated by introducing the construct pME6010-*bceQ*full into Δ*bceQ* mutant. pME6010-*bce*Qfull was made by cloning a 1,540 bp-DNA fragment which contains the *bceQ* full gene into pME6010 at the *Xho*l and *Hind*III restriction enzyme sites. The complementary strain Δ*bce*Q-Comp was confirmed by PCR analysis (Supplementary Figure S1). Strains LK749 and LK750 were made by introducing the empty vector pME6010 into the wild type PsJN and the Δ*bceQ* mutant, respectively.

### EPS production assay

PsJN and its derivatives were cultured in ½ PDB liquid medium at 28°C with shaking for 3 days. The cultures were centrifugated for 5 min at 8,000 r.p.m. to remove the cells and collect the supernatant. To extract the EPS, pure ethanol proof 200 was mixed with the culture supernatant at a ratio of 2:1 (ethanol: supernatant, v/v). The EPS was precipitated by incubating the mixtures at −20°C overnight and centrifugated for 10 min at 12,000 r.p.m. The clear supernatants were carefully discarded, and the pelleted EPS was dried at 45°C overnight, weighed, and the dry weight of EPS was normalized by the volume of the bacterial cultures. The experiment was repeated three times independently.

### Bacterial motility assay

Bacterial motility is important in the interaction with the plant host and can be influenced by EPS production. The swimming and swarming motility assays were conducted on 0.2% agar and 0.6% agar of TSA plates, respectively. Specifically, 2 μl of the bacterial cell suspension (OD_600_ = 0.1, equals around 10^8^ cfu/ml) were inoculated on the fresh media plates. The plates were incubated at 28°C without disturbance for 3 days before the diameters (cm) of the spreading colonies were measured. The experiment was repeated two times independently with three replicates of all the tested strains in each experiment.

### Growth assays in culture media

PsJN may encounter salt and/or iron stresses in environments because of the accumulation of different salts *via* natural processes and agricultural practices (Kumar et al., 2020). To test the bacterial resistance to salt and iron stresses, NaCl and FeCl_3_ were added to the KB liquid medium at a final concentration of 100 and 1 mM, respectively, based on our preliminary experiments. The growth assays were conducted in 96-well plates. Specifically, the bacterial cells of PsJN and the Δ*bceQ* mutant were inoculated in KB broth (with and without salt amendments) at a starting OD_600_ = 0.001 (around 10^6^ cfu/ml). A 250 μl of the starting cultures was aliquoted into a 96-well plate with each strain having four replications. The 96-well plate was incubated in a SPARK® multimode Microplate Reader (TECAN, Switzerland) with continuous shaking (around 200 r.p.m.) at room temperature. The cell density was measured every 2 h by recording the absorbance of optical density at 600 nm. All the growth assay experiments were conducted two times independently, with each experiment including four replicates of the tested strains.

### Resistance assays to desiccation and UV (ultraviolet light) stress

Wild type PsJN and the Δ*bceQ* mutant were inoculated on sterilized filter papers (Millipore, 0.45 μm, Bedford Massachusetts) that were placed on ½ PDA plates. The plates were incubated at 28°C for 3 days before the following desiccation or UV stress assays. For the desiccation resistance assay, the bacterial colonies, carried by the filter papers, were removed from the culture plates and dried in a sterilized Petri dish at 28°C. After drying, the population size of the bacterial colonies (cfu/colony) was measured immediately (day 0), and on 2, 8, and 14 days. Specifically, the filter paper carrying bacterial colonies was cut into small pieces with one colony per piece of the filter paper. The population size of the colonies was measured by placing one piece of filter paper in 1 ml of sterilized water followed by serial dilution plating on PDA agar plates. For the UV stress resistance assay, the bacterial colonies were removed from the culture plates and placed in a sterilized Petri dish as described above. The colonies were directly exposed to UV radiation in a lumina flow hood (Purifier Logic+ Class II, Type A2 Biosafety Cabinet, Labconco Corporation). The bacterial colonies were sampled at 0, 10, 30, and 50 min after the UV exposure and the population size (cfu/colony) was measured by dilution and plating assay as described above. Both the desiccation and the UV resistance experiments were repeated three times independently with each experiment having four replicates of the tested strains at each time point.

### Bacteriophage tolerance assay

The bacteriophage tolerance assay was conducted by following the previous report with modifications (Maynard et al., 2010). The bacteriophage vB_Pap-BS21 (*Paraburkholderia phytofirmans* strain PsJN, Bozeman soil, 2021) was isolated from the greenhouse pot soils following the previous report (Fu et al., 2021). The phage was purified through five rounds of plaque purification on bacterial lawn plates. To test the impact of the isolated bacteriophage on the growth of PsJN, the same volume of the bacterial cell suspension (OD_600_ = 0.1, equals around10^8^ cfu/ml) was mixed with the phage particle suspension (10^6^ pfu/ml) to reach a ratio of phage particle: bacterial cell equals 1:100 in ½ PDB liquid medium. The mixtures were aliquoted into a 96-well plate with 250 μl per well. The 96-well plate was incubated in the Tecan Microplate Reader with continuous shaking at room temperature and the cell density was recorded by measuring the absorbance of optical density at 600 nm. The experiments were repeated two times independently, with each experiment included eight replicates of the tested strains.

### Transmission electron microscopy analysis

TEM was used to examine bacterial cell morphology. Bacterial cells that were collected from fresh cultures on KB plates were negatively stained using the negative stain solution 1% phosphotungstic acid before the TEM analysis. The experiment was conducted at the TEM Center of Montana State University.

### Plant colonization assay

Camelina (*Camelina sativa* cv. Suneson) and common pea (*Pisum sativum*) supported a decent endophytic colonization by PsJN in our preliminary experiments and were used in this study. The seeds were sowed in 6-inch pots containing soils (pH: 7.3; NO_3_-N: 68 ppm; PO_4_-P: 3 ppm; potassium: 47 ppm) that were autoclaved twice at 121°C for 2 h. The pots were kept in a greenhouse that was maintained at 22/18 ± 1°C (day/night), a humidity level of 42%, and a photoperiod of 16/8 h light/darkness, at the Plant Growth Center, Bozeman, Montana. Regular watering was provided based on the moisture level of the soil. Drought stress, if needed, was introduced by stopping watering the plants immediately after the bacterial inoculation (as described below). The plant samples were collected and analyzed on day 7 after inoculation when the drought-induced wilting symptoms were visibly shown.

Leaves of four-week-old camelina and three-week-old pea were inoculated with the bacterial strains and tested for their plant colonization. Specifically, the bacterial suspension (OD_600_ = 0.1) was injected into leaves using a needleless syringe until the inoculation covered most areas of the leaf. Each bacterial strain was tested on three plants, with each plant having three leaves treated. The bacterial colonization was enumerated on days 0 and 7 by serial dilution plating of the extracts of a leaf disc (around 1 cm^2^) onto KB plates added with Rifampicin (100 μg/ml). The plates were incubated at 28°C until colonies developed, which typically took 3 days.

### Statistical analysis

Statistical analysis was performed using the Samples *t*-test function of the SPSS software. A *p*-value of less than 0.05 was considered a significant difference. The sample sizes and number of replicates of each specific experiment were indicated in the respective methods described above.

## Results

### Mutation of Δ*bceQ* abolished EPS production

**Figure 1:**
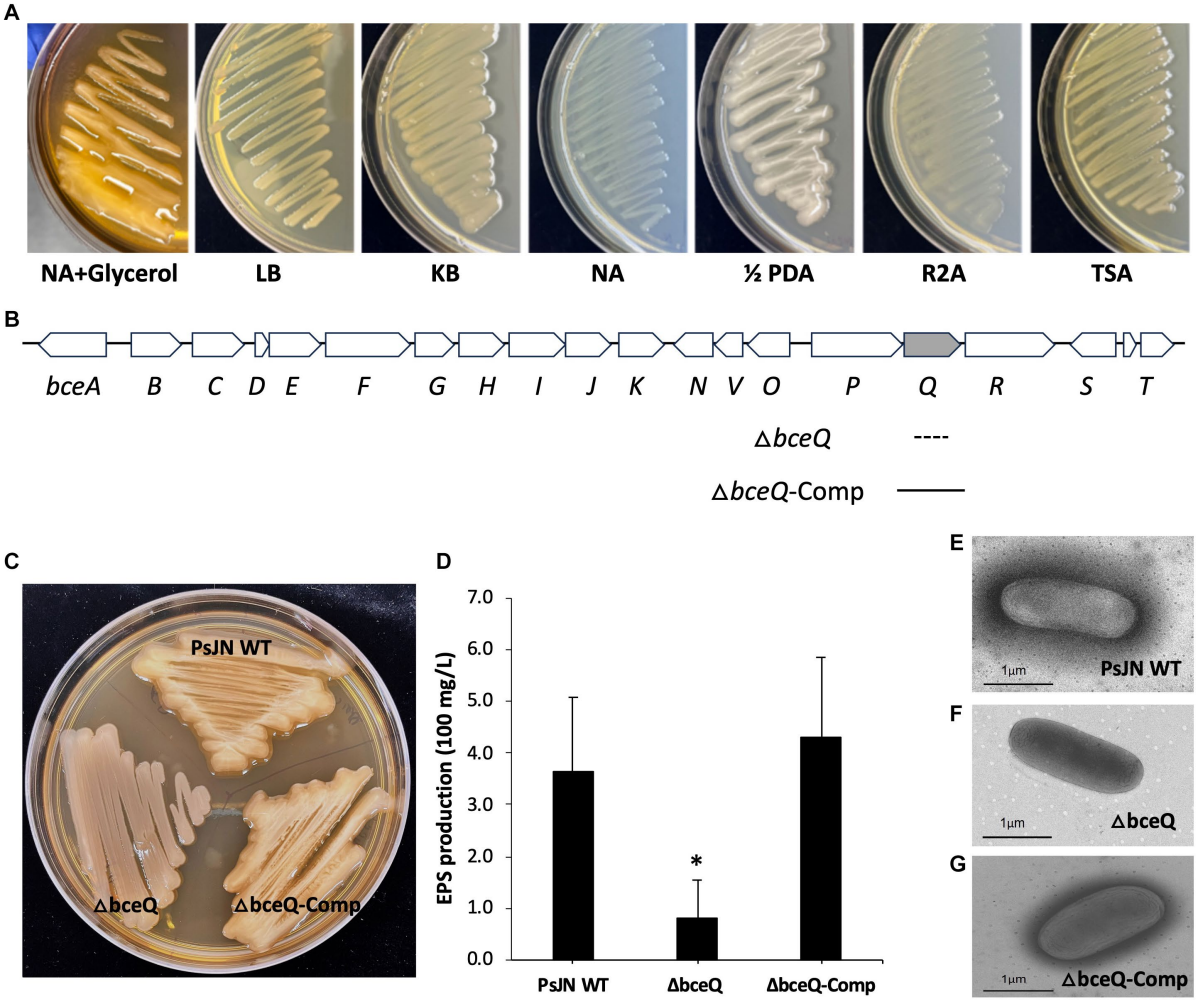
Characterization the biosynthesis gene *bceQ* in EPS production of *P. phytofirmans* PsJN. **(A)** Colony morphology of PsJN on seven different culture media. Colonies that were more mucoid indicate a higher level of EPS production. **(B)** The EPS biosynthesis gene cluster of PsJN. The DNA fragment that was deleted in the mutant and the DNA fragment that was used in the complementation strain was indicated as a dotted line and a filled line, respectively. **(C)** Colony morphology of PsJN and its derivatives on NA + Glycerol plates. PsJN WT: wild type PsJN containing empty vector pME6010; Δ*bceQ*: Δ*bceQ* mutant containing empty vector pME6010; Δ*bceQ*-Comp: Δ*bceQ* mutant containing the complementary plasmid pME6010-bceQFull. **(D)** Quantification of EPS production of PsJN in ½ PDB liquid cultures. Data represent the means of results from three independent experiments. Error bars show standard deviations. * Indicates significant difference, as determined by *t*-test (*p* < 0.05). **(E–G)** TEM analysis of PsJN cells. Bacterial cells were collected from a fresh culture plate without washing and observed under TEM.

PsJN can grow on different media although the biosynthesis of EPS might be favorited under specific culture conditions. To identify the culture media that favorite the EPS production, the wild type PsJN was cultured on agar plates that were made of seven different culture media, including NA, NA + Glycerol, LB, KB, half strength (½) PDA, R2A, and TSA. As shown in Figure 1A, PsJN formed more mucoid colonies, indicating a higher level of EPS production, on NA + Glycerol and ½ PDA plates than the other tested media. Further, PsJN grew slightly faster on ½ PDA than on NA + Glycerol (data not shown). Thus, the ½ PDA medium was used in the following EPS assay.

The genome of PsJN contains a cluster of 19 genes that encode proteins that are putatively responsible for the biosynthesis, translocation, and export of EPS (Figure 1B). To characterize the role of the EPS biosynthesis genes in EPS production, an in-frame deletion of *bceQ*, encoding a putative flippase for the translocation of EPS intermediates from inner membrane to periplasmic spaces, was introduced in the genome of wild type PsJN (Figure 1B; Supplementary Figure S1). The resultant Δ*bceQ* mutant formed much less mucoid colonies than the wild type PsJN on culture plates (Figure 1C). The complementary strain Δ*bceQ*-Comp, containing the wild type *bceQ* gene on a plasmid, restored the colony morphology similar to the wild type strain. To quantify the EPS production, PsJN and its derivatives were cultured in ½ PDB liquid medium, and the produced EPS was extracted and measured. As shown in Figure 1D, the Δ*bceQ* mutant produced EPS at a significantly lower level than the wild type and the complementary strain (Figure 1D), thus validating the role of *bceQ* in the EPS production of PsJN. Interestingly, a dark layer of the extracellular substrate was observed surrounding cells of the wild type PsJN, the complementary strain, but not the Δ*bceQ* mutant (Figures 1E–G), which is consistent with the known function of EPS as the extracellular polysaccharide on the outside surfaces of the producing cells.

### Lack of EPS production affected bacterial motility on culture plates

**Figure 2:**
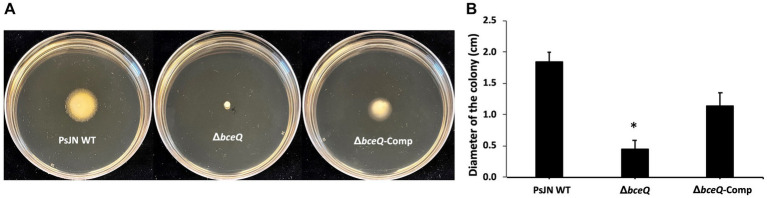
Motility assays of PsJN on culture plates. **(A)** The swimming motility of Δ*bceQ* mutant decreased significantly compared to wild type and complementary strain on TSA plates containing 0.2% agar. **(B)** The swimming motility was determined by measuring the bacterial colony size (diameter, cm) on the plates on day 4 after inoculation. PsJN WT: wild type PsJN containing empty vector pME6010; Δ*bceQ*: Δ*bceQ* mutant containing empty vector pME6010; Δ*bceQ*-Comp: Δ*bceQ* mutant containing the complementary plasmid pME6010-bceQFull. Data represent the mean values of three repeats. Error bars show standard deviations. * Indicates significant difference, as determined by *t*-test (*p* < 0.05). The experiments were repeated three times with similar results.

PsJN and its derivatives were cultured on TSA plates that contained 0.2 and 0.6% of agar to assess the swimming motility and swarming motility, respectively. The results showed that the Δ*bceQ* mutant had a significantly decreased swimming motility, as determined by the decreased diameter size of the bacterial colony spreading on the culture plates, than the wild type and the complementary strains (Figure 2). No significant difference of the swarming motility was observed between the wild type PsJN and the Δ*bceQ* mutant (data not shown). This result suggests that EPS production positively influenced the swimming motility of PsJN. This result is consistent with the previous reports that EPS, similar to other polysaccharides, may serve as motility substrates facilitating bacterial movement (Gygi et al., 1995; Berleman et al., 2016).

### The EPS production was required in the tolerance to salt and iron stresses

**Figure 3:**
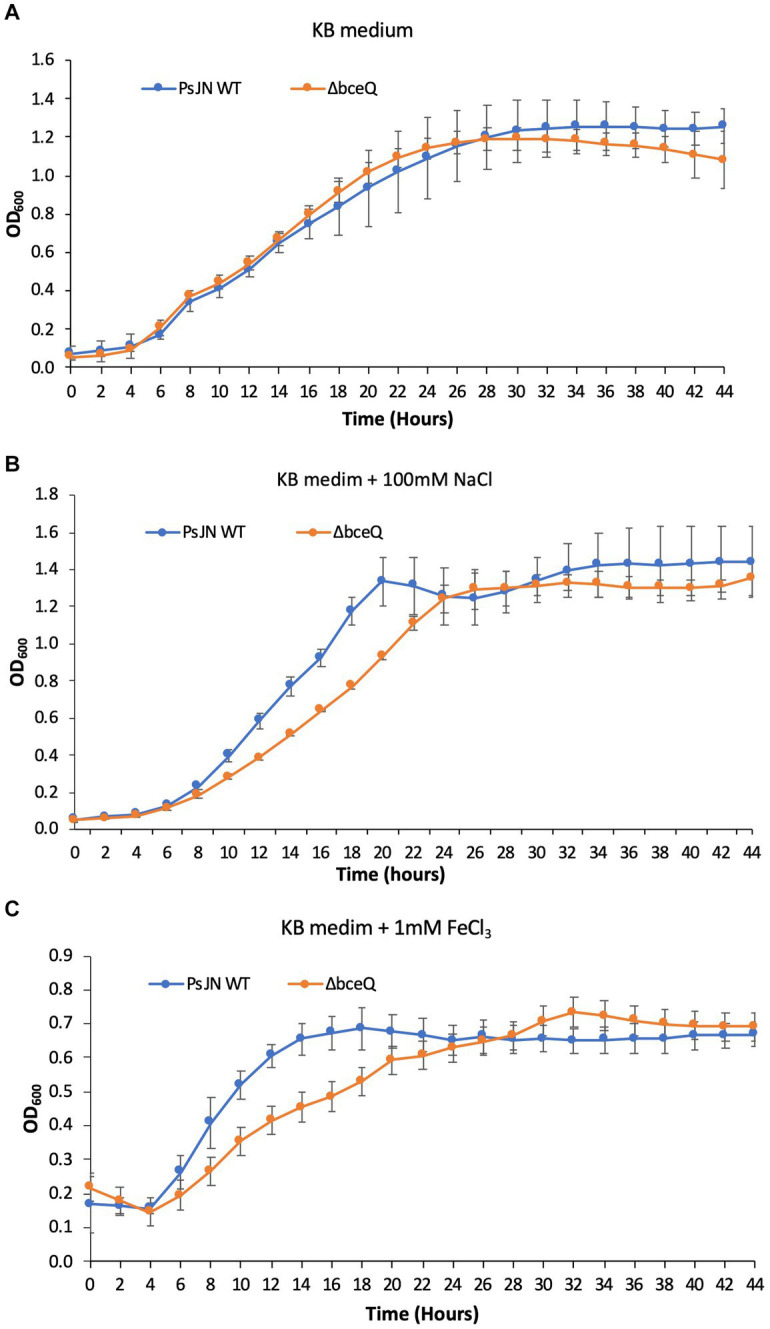
Growth assays of PsJN and its derivates in culture media. The wild type PsJN and the Δ*bceQ* mutant were cultured in KB only **(A)** KB amended with 100 mM NaCl **(B)** and KB amended with 1 mM FeCl_3_ **(C)**. The bacterial growth was measured by recording the OD_600_ values of the cultures using a 96-well plate reader. Data represent the mean values of at least three repeats. Error bars show standard deviations. The experiments were repeated two times independently.

To investigate if lack of EPS production affects PsJN’s growth under normal culture conditions or with the presence of salt and iron stress, the wild type PsJN and the Δ*bceQ* mutant were cultured in KB broth with and without the addition of NaCl and FeCl_3_. As shown in Figure 3A, the Δ*bceQ* mutant had a similar growth curve as the wild type PsJN, indicating that lack of EPS production does not affect the growth of PsJN in the KB medium.

Adding NaCl at 100 mM did not influence the wild type PsJN’s growth but negatively affected the Δ*bceQ* mutant with a prolonged exponential growth stage (Figure 3B), indicating a decreased growth rate of the mutant under the salt stress. The addition of FeCl_3_ at 1 mM decreased the growth of the wild type PsJN and the Δ*bceQ* mutant to a maximal OD6_00_ of 0.8 (Figure 3C), which is lower than their maximal OD6_00_ of 1.2 in KB without treatment. Importantly, the Δ*bceQ* mutant showed a clearly prolonged exponential growth profile, compared to the wild type PsJN, with the FeCl_3_ treatment (Figure 3B).

Overall, these results suggested that EPS production was required for the maximal growth of PsJN under the salt and iron stresses.

### EPS plays a role in PsJN tolerance to desiccation and UV damage

**Figure 4:**
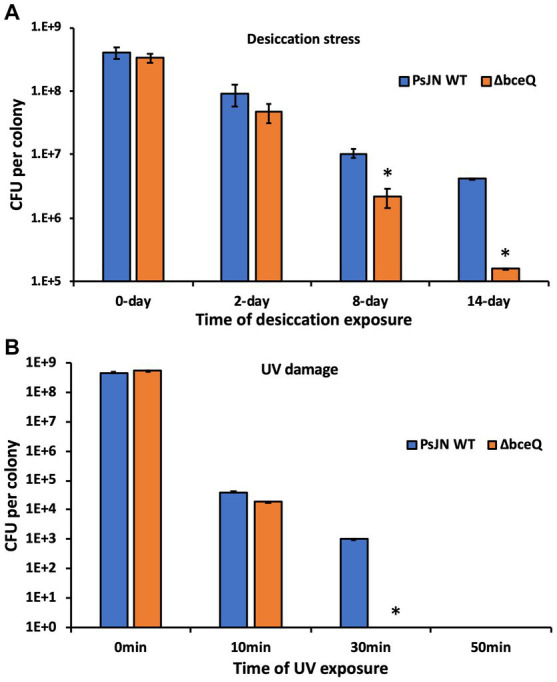
Survival assays of PsJN and its derivatives under desiccation **(A)** and UV damage **(B)** stresses. Colonies of the wild type PsJN and the Δ*bceQ* mutant were cultured on filter papers on top of a ½ PDA plate. The bacterial colonies were cultured for 3 days, removed from the culture plates, and used in the desiccation assay **(A)** and the UV stress assay **(B)**. Data represent the mean values collected from a total of 12 colonies (4 colonies per filter paper × 3 filter papers per strain) for the tested strain at each time point. Error bars show standard deviations. * Indicates significant difference, as determined by *t*-test (*p* < 0.05). The experiments were repeated two times independently.

To test if EPS protects PsJN from desiccation and UV damage, the wild type PsJN and the Δ*bceQ* mutant were inoculated on filter papers that were placed on the top of ½ PDA plates. The colonies were removed on day 3 and dried in an empty petri dish to test the bacterial survival under desiccation. As shown in Figure 4A, the populations of both wild type PsJN and Δ*bceQ* mutant decreased with the extended desiccation. However, the Δ*bceQ* mutant’s population declined faster than the wild type. For example, around 1% of the wild type PsJN’s population remained alive on day 14, but only less than 0.1% of the Δ*bceQ* mutant’s population survived at this time point.

To test the bacterial survival under UV stress, the above cultured fresh bacterial colonies were placed under a UV light, and the living cells were measured at different time points. The results showed that no living cells of the Δ*bceQ* mutant were detected at 30 min after UV exposure, but around 0.01% of the wild type’s population remained alive (Figure 4B).

These results suggested that EPS production was required by PsJN to tolerate desiccation and UV damage.

### The Δ*bceQ* mutant was more sensitive to bacteriophage infection

**Figure 5:**
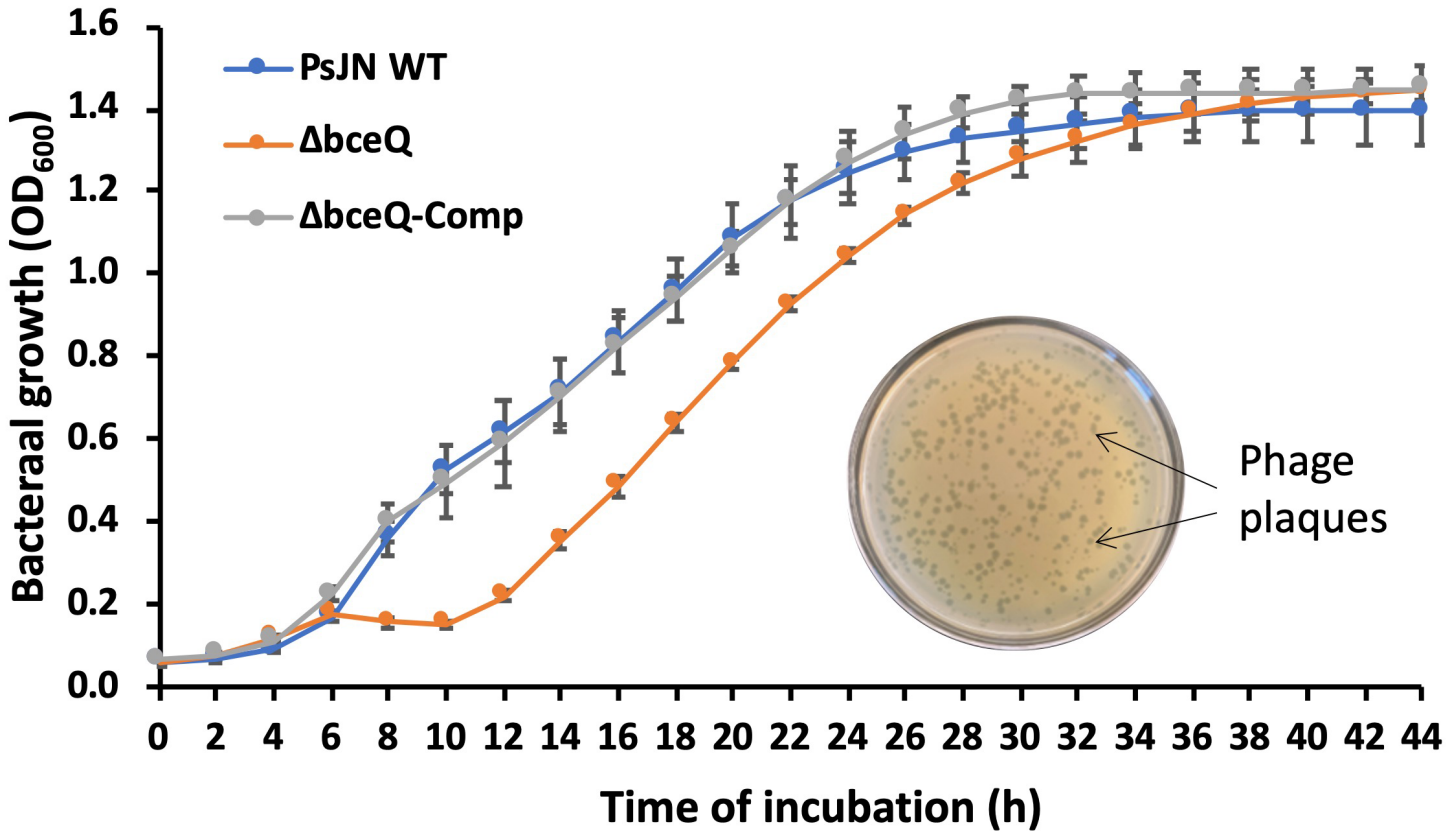
Infection assays of PsJN and its derivative by bacteriophage. Inner photo shows the transparent phage plaque formed on the bacterial lawn of wild type PsJN infected by the phage vB_PapBS21. The bacterial growth was measured by mixing the bacteriophage particles with bacterial cells at a ratio of 1:100 (phage particle:bacterial cell) in ½ PDB liquid medium. The bacterial growth was measured by recording the OD_600_ values of the cultures using a 96-well plate reader. PsJN WT: wild type PsJN containing empty vector pME6010; Δ*bceQ*: Δ*bceQ* mutant containing empty vector pME6010; Δ*bceQ*-Comp: Δ*bceQ* mutant containing the complementary plasmid pME6010-bceQFull. Data represent the mean values of at least three repeats. Error bars show standard deviations. The experiments were repeated two times.

Bacteriophages are bacterial natural enemies that are ubiquitous in the environment and can affect bacterial survival and activity. In this work, a bacteriophage vB_Pap-BS21 isolated from greenhouse pot soils was found to infect PsJN and caused transparent plaques on culture plates (Figure 5, inner photo). To test if EPS plays a role in protecting PsJN cells against phage infection, the purified bacteriophage particles were inoculated in the bacterial cultures of the wild type PsJN and the Δ*bceQ* mutant. Inoculation of the bacteriophage caused a clear growth deficiency at the early exponential stage of the Δ*bceQ* mutant, compared to the wild type PsJN and the complementary strain (Figure 5). All the tested strains eventually reached the stationary phase, suggesting that the phage DNA probably integrated into the bacterial genome and became a prophage. However, the compromised growth of the Δ*bceQ* mutant at the exponential stage by the phage treatment indicated that EPS likely protected PsJN from bacteriophage infection at the early stage of the bacterial growth.

### EPS production was required in endophytic colonization of PsJN

**Figure 6:**
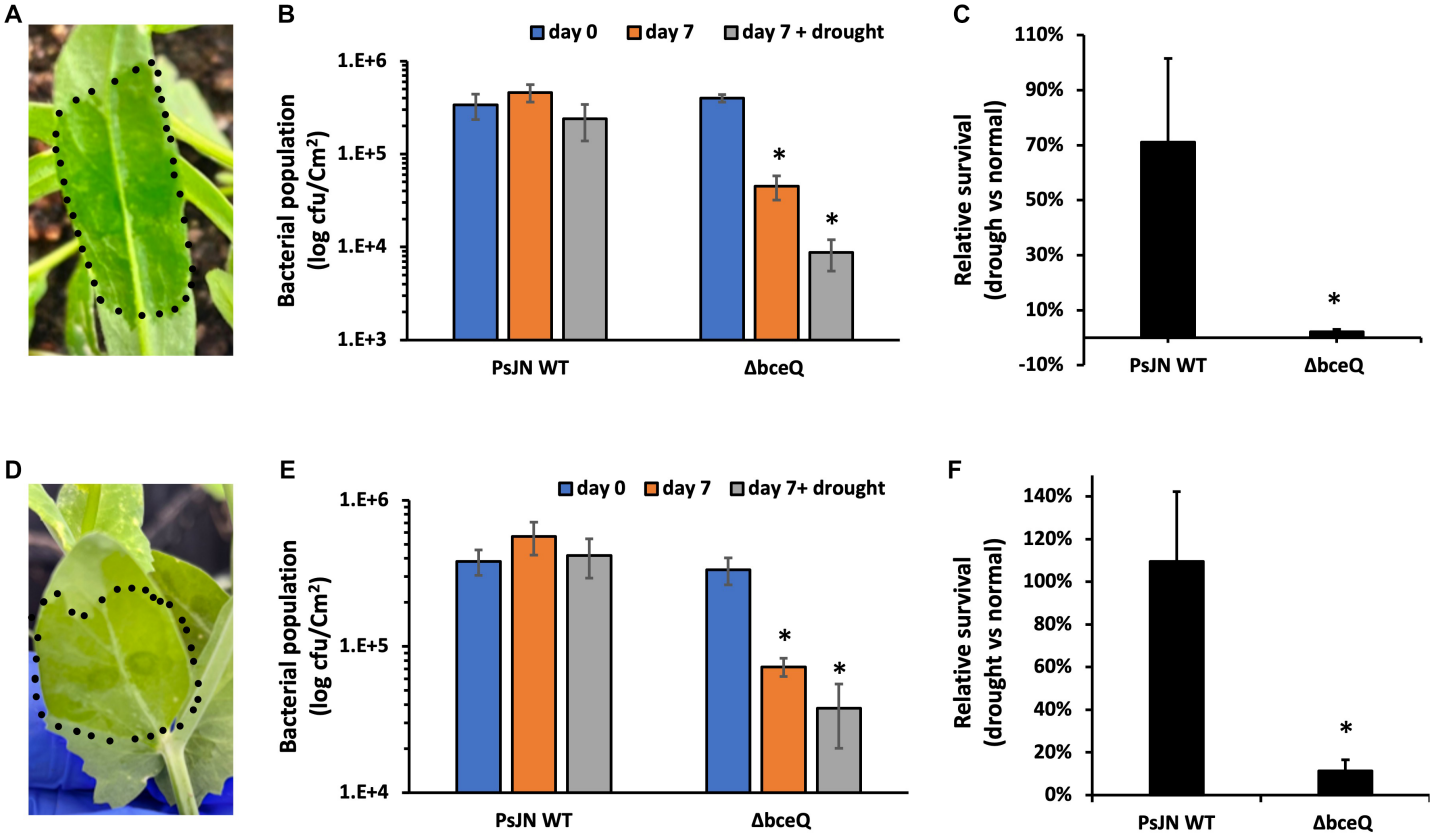
Colonization assays of PsJN and its derivatives in leaves of camelina (*Camelina sativa* cv. Suneson) **(A–C)** and pea (*Pisum sativum*) **(D–F)**. **(A,D)**, Leaves were infiltrated by bacterial suspension using a needleless syringe. The inoculation area was indicated by the dotted line. **(B,E)** Bacterial populations were recovered from leaves and enumerated on days 0 and 7 by serial dilution plating of the leaf extracts onto KB plates added with Rifampicin (100 μg/ml). **(C,F)** Relative bacterial population on day 7 under plant drought stress compared with their populations under normal growth conditions. The plant drought stress, indicated by wilting leaf symptoms, was induced by stopping watering for 7 days immediately after the bacterial inoculation. Data represent the mean values of at least three repeats. Error bars show standard deviations. The experiments were repeated two times. * Indicates significant difference, as determined by *t*-test (*p* < 0.05).

Camelina and common pea were used to test the role of EPS in the endophytic colonization of PsJN. The wild type PsJN and the Δ*bceQ* mutant were injected into the leaves using a needleless syringe (Figures 6A,D). The bacterial populations were measured on day 0 and day 7 after inoculation. As shown in Figures 6B,E, the wild type PsJN and the Δ*bceQ* mutant had a similar level of bacterial population size on day 0. However, the Δ*bceQ* mutant’s population was significantly lower than the wild type PsJN on day 7 after inoculation in both plants, indicating that EPS production is important for a successful colonization of PsJN in the tested plants.

Further, we tested the bacterial colonization under plant drought stress by stopping watering immediately after the bacterial inoculation. As shown in Figure 6C, around 70% of the wild type’s population survived on day 7 under the drought stress of camelina plant relative to the bacterial population on day 7 under the normal plant growth condition. However, only less than 10% of the Δ*bceQ* mutant’s population survived under the plant drought stress relative to the normal plant growth condition. A similar result was also observed when the strains were tested in pea leaves (Figure 6F).

Overall, these results showed that EPS production was required for the endophytic colonization of PsJN in camelina and pea under not only the normal growth condition but also the drought stress of the host plants.

## Discussion

EPS is known to play an important role in the colonization of endophytic bacteria on host plants. *P. phytofirmans* PsJN has served as a model strain to study the molecular mechanisms of plant-endophytic bacteria interactions. Although genomic analysis has revealed that PsJN contains a cluster of 19 genes that putatively contribute to the EPS biosynthesis (Ferreira et al., 2010; Weilharter et al., 2011), their functions in the EPS production of PsJN have not been validated and the role of EPS production in plant-PsJN interactions remains uncharacterized. We addressed these questions by creating an EPS mutant that lacks the *bceQ* gene and characterizing the Δ*bceQ* mutant’s tolerance to different biotic and abiotic stresses and plant colonization in two different crops including camelina and pea.

Gene *bceQ* encodes a putative flippase to translocate the EPS intermediates oligosaccharide across the inner membrane (Ferreira et al., 2010). Here, we created a Δ*bceQ* mutant which has an in-frame deletion of the *bceQ* gene in PsJN. The Δ*bceQ* mutant produced a significantly decreased level of EPS production on culture plates and in liquid broth (Figures 1C,D). It was reported that mutation of *bceQ* gene abolished cepacian production of *Burkholderia cepacia* IST408 (Ferreira et al., 2010). Further, a *bceN* gene, which locates upstream of *bceQ* in the *bce* gene cluster, was recently found to be involved in the cepacian production of a closely related species *P. phymatum* (Liu et al., 2020). Thus, our results are consistent with these previous reports and provided genetic evidence to confirm that the *bce* gene cluster is responsible for the major EPS production in strain PsJN.

EPS production of PsJN plays an important role in the tolerance to various biotic and abiotic stresses including desiccation, UV damage, salt and iron stresses, and bacteriophage infection. These environmental stresses are the harmful factors that endophytic bacteria may constantly be exposed to during the interactions with host plants and can challenge their plant colonization (Pfeilmeier et al., 2016). The roles of EPS production in the resistance of PsJN to environmental stresses are consistent with the known functions of EPS in bacterial survival, colonization, and stress alleviation (Costa et al., 2018). For example, strains of the *Burkholderia* species were found to better withstand desiccation stress with the presence of 2.5 g/liter of cepacian (Ferreira et al., 2010). The role of EPS in desiccation tolerance is likely due to the highly charged nature of EPS which aids in water absorption. PsJN has been reported to promote the growth of wheat, maize, and potato under drought conditions (Naveed et al., 2014, 2015; Sheibani-Tezerji et al., 2015). Additionally, PsJN can also improve salt stress resistance of different plants including quinoa and *A. thaliana* (Pinedo et al., 2015; Yang et al., 2020). Similarly, *Paraburkholderia* sp. GD17 was reported to improve rice seedlings’ tolerance to salt stress, possibly *via* the absorption and redistribution of mineral elements Na^+^ in the apoplast (Zhu et al., 2021). It will be interesting to investigate if EPS production plays a role in the PsJN-mediated plant resistance against drought and salt stresses in future research. Relative to bacterial growth with the NaCl (100 mM) treatment, PsJN showed a more compromised growth with the presence of FeCl_3_ even at a low concentration of 1 mM (Figure 3). The stress is likely caused by Fe^3+^ but not Cl^−^ because the added Cl^−^ was at a concentration of 3 mM which is much lower than the NaCl treatment that did not affect the PsJN’s growth even at the concentration of 100 mM (Figures 3A,B). The FeCl_3_ treatment probably posed an oxidative stress to the bacterial cells, because excessive iron is known can be harmful to cells by generating reactive oxygen species *via* the Fenton reaction (Giannakis et al., 2017).

Bacteriophages are natural enemies of bacteria and can threaten bacterial survival and activity. EPS capsule is known to function as a normal physical barrier to phages’ infection (Born et al., 2014). Decreased phage attachment to the targeted bacterial cell surface was observed with increased EPS production (Liu et al., 2017). Consistent with these reports, mutation of EPS biosynthesis gene *bceQ* led to a compromised growth of PsJN with the presence of bacteriophage vB_Pap-BS21 (Figure 5), supporting that EPS plays a role in the resistance to the phage infection. Spontaneous PsJN mutants that were resistant to the bacteriophage infection were identified (Fu and Yan, unpublished result). Future research is needed to test if these mutants increase bacterial survival and activity in plant-bacteria interactions with the presence of bacteriophage.

Mutation of EPS biosynthesis gene *bceQ* significantly decreased the endophytic colonization of PsJN under both normal growth and plant drought stress conditions (Figure 6). A successful plant colonization is required for the endophytic bacteria to confer the beneficial effects on plant growth and health. PsJN is known to successfully colonize a wide range of plant species, which was attributed to its large genome (8.2 Mbp and 7,484 genes) that harbors a diverse array of physiological functions (Mitter et al., 2013), although the underlying molecular mechanisms remain largely uncharacterized. Knockout of *bpl.1*, encoding a signal synthase of the quorum sensing regulatory system of PsJN, caused lower endophytic colonization in *A. thaliana* (Zúñiga et al., 2013). Interestingly, expression of the EPS biosynthesis gene *bceB* (Bphyt_1955) was downregulated in the Δ*bpl.1* mutant compared to the wild type PsJN (Zúñiga et al., 2013), indicating that the downregulated EPS production probably led to (at least partially) the lower level of plant colonization by the Δ*bpl.1*mutant. This report is consistent with the previous hypothesis that the production of EPS might be involved in the plant-PsJN interaction (Ferreira et al., 2010). In this study, we found that the Δ*bceQ* mutant had a significantly lower level of endophytic colonization than the wild type PsJN in camelina and pea plants (Figure 6), providing strong genetic evidence to support that EPS production indeed plays an important role in the plant colonization of PsJN.

Compared to the opening plant epiphytic environments, plant interiors likely provide a more stable environment for the endophytic bacteria to colonize and survive. However, the plant interior is also constantly changing because of the physiological adaptions to plant development and plant responses to environmental stress. For example, plants increase cellular osmotic adjustment, *via* the accumulation of organic and inorganic solutes, as a prime adaption strategy to tolerance drought stress (Blum, 2017). The changed plant interior environment can pose challenges for bacterial endophytic colonization. Successfully maintaining the colonization under plant stress condition is critical for the endophytic bacteria to provide benefits to the hosts. However, very little is known about the molecular mechanism of how endophytic bacteria survive the interior of plants under stressful conditions. Nevertheless, a recent transcriptomic analysis of PsJN colonized in potato under drought stress revealed that hundreds of genes were differentially regulated to adapt to the changes when colonized in the plant (Sheibani-Tezerji et al., 2015). Interestingly, plant drought stress activated the expression of *bceA*, *bceC*, *bceQ,* and *bceT* of PsJN in at least one of the three tested time points (1 h, 6 h, and 12 h of drought stress treatment), implying that PsJN upregulated the EPS biosynthesis genes to cope with the plant drought stress. This hypothesis was supported by our data that mutation of *bceQ* significantly reduced the endophytic colonization of PsJN in the leaves of camelina and pea under drought conditions (Figure 6). To the best of our knowledge, this is the first report that EPS biosynthesis gene is required for the successful colonization of endophytic bacteria in the plant tissues under stress conditions.

## Conclusion

This study characterized the EPS biosynthesis gene *bceQ* and the functions of EPS production in plant-bacteria interaction of the model endophytic bacterium *P. phytofirmans* PsJN. Our results confirmed the function of the *bce* gene cluster in the EPS biosynthesis of PsJN. Further, we showed that EPS production plays an important role in the tolerance of PsJN against various biotic and abiotic stresses inducing desiccation, UV damage, salt and iron stresses, and bacteriophage infection, and is required for successful endophytic colonization in camelina and pea under normal growth and plant drought stress condition.

## Data availability statement

The raw data supporting the conclusions of this article will be made available by the authors, without undue reservation.

## Author contributions

BF and QY conceived the project. BF conducted the experiments and wrote the manuscript. QY revised and approved the manuscript. All authors contributed to the article and approved the submitted version.

## Funding

This research was supported by the U.S. Department of Energy, Office of Science, grant no. DE-SC0021369.

## Conflict of interest

The authors declare that the research was conducted in the absence of any commercial or financial relationships that could be construed as a potential conflict of interest.

## Publisher’s note

All claims expressed in this article are solely those of the authors and do not necessarily represent those of their affiliated organizations, or those of the publisher, the editors and the reviewers. Any product that may be evaluated in this article, or claim that may be made by its manufacturer, is not guaranteed or endorsed by the publisher.
