# Supplementary figures and images for: Exopolysaccharide is required for motility, stress tolerance, and plant colonization by the endophytic bacterium Paraburkholderia phytofirmans PsJN
Source: Front Microbiol. 2023 Aug 21;14:1218653. doi: 10.3389/fmicb.2023.1218653 (PMC10475733; doi:10.3389/fmicb.2023.1218653)

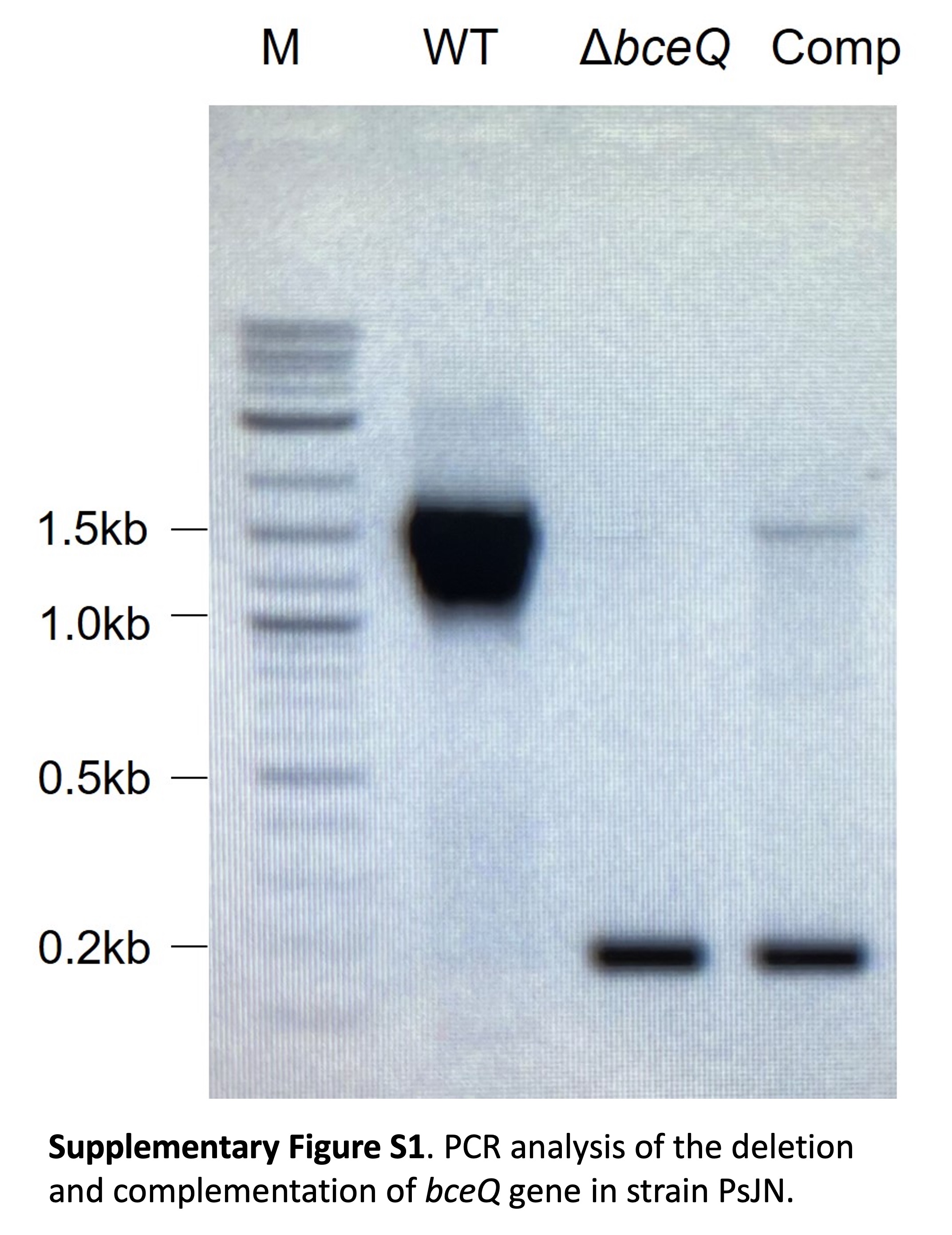

Supplement: Supplementary file 1 [file Image_1.JPEG]
